# Art and Disease

**Published:** 1894-01-20

**Authors:** 


### Art and Disease.

II.—POSSESSION OR HYSTERIA *(continued).*

From an examination of some early works of art it has been seen that the phenomena, classed in the Middle Ages under the title of demoniacal possession, presented many points of resemblance to hysteria. Advancing to the time of the Renaissance the pathological interest in the delineation of the "possessed" grows stronger. A somewhat detailed description of a fresco of Andrea del Sarto in the Annunziata at Florence will not be out of place. The subject is Saint Philip de Néri delivering a woman possessed by a demon. The figure of the young woman has been greatly admired. Charles Blanc in his "History of Painters" remarks that "she faints with an involuntary grace and such exquisite truth, that the greatest masters might have wished to have created the form." Side by side with this criticism of the artist it is instructive to have the criticism of the physician.* The admiration of M. Charcot is not less fervent for being more discriminating. He finds that the picture was indisputably painted from life. "Seized by the disease, the young woman falls backward, and the rigidity of the body has already set in. This fall has nothing of the self-surrender, with muscular flaccidity of syncope or a faint, as Charles Blanc imagined. One perceives that her body, curved thus backwards, is stiff from head to foot. The lower limbs, slightly bent, are contracted, as witness the convulsion of the feet, with toes turned inwards. The head, violently thrown back, forces into prominence the swollen neck, and the turgescent, puffed-out face betrays the stoppage of the respiration, brought about by the generalised spasm. The arms are being thrust out as though to commence the wild tonic movements, which the two assistants seem to restrain. It is true that, according to our hypothesis, the fingers should be clenched in the palm of the hand and the fore-part of the arms turned downwards."

By far the most familiar representation of a demoniac is that of Raphael in the picture of the "Transfiguration." Sir Charles Bell was, perhaps, the first to detect the insincerity of the symptoms exhibited. He says of it†: "A physician will conclude that this youth was feigning. He is, I presume, convulsed; he is stiffened with contractions, and his eyes turned in their sockets. But no child was ever so affected. In real convulsions the extensor muscles yield to the more powerful contractions of the flexor muscles; whereas in the picture the lad extends his arms, and the fingers of the left hand are stretched unnaturally backwards. Nor do the lower extremities correspond with truth; he stands firm; the eyes are not natural; they should have been turned more inwards, as looking into the head, and partially buried under the forehead. The mouth, too, is open, which is quite at variance with the usual condition." Of the attitude of the hand M. Charcot observes that among the attitudes, varied in a thousand ways, which are assumed during a convulsive attack, this is perhaps the only one he has never noticed. These criticisms may be verified by anyone possessing a good photograph of this masterpiece. It is hard to account for the facts short of the hypothesis that Raphael employed for his model a healthy youth who was directed to throw himself into the required attitude, and who was, as Sir Charles Bell suggests, actually feigning,

The Flemish painter, Déodat Delmont, who imitated Raphael minutely as regards the composition of his "Transfiguration," resolutely forsook his example in the rendering of his demoniac. His picture shows a study of the convulsions of hystero-epilepsy, drawn evidently from life. Raised from the ground by a powerful man behind, who is evidently exerting his full strength to restrain him, the boy is struggling wildly in the convulsions of "clownism." With one clenched fist he threatens to strike all who approach. With the other hand he strives in vain to tear off his clothes. The convulsed eyeballs reveal inward strabism, and the mouth is half open in an accurately noted convulsive attitude. Whether the effect from an artistic point of view is more pleasing is another question.

The demoniac painted by Carracci's pupil, Dominichino, though a far less ambitious study, has been recognixed both by Sir Charles Bell and by M. Charcot as infinitely more true to nature. It is true that these authorities differ in the interpretation of the symptoms. Sir Charles Bell traces them to the opisthotonic spasm of tetanus, but finds them not altogether consistent. "Convulsions have seized the lad; he is rigidly bent back, the lower limbs spasmodically extended, so that his toes only rest on the ground; the eyes are distorted and the pupils turned up under the eyelids. This would be the position of opisthotonos, were not the hands spread abroad, the palms and fingers open, and the jaw fallen. Had the representation been perfectly true to nature, the jaws would have been clenched and the teeth grinding." M. Charcot, seeing hysteria rather than tetanus indicated by the symptoms, argues that the open mouth and outstretched arms are frequent accompaniments to the posture of "arc de cercle," which the lad has assumed; but he agrees with Sir Charles Bell as to the inconsistency of the open palms of the hand. With the exception of this trifling error, Dominichino has succeeded in this wonderfully accurate representation to the admiration of physicians and artists alike.

Rubens, for whom the subject of demoniac possession offered apparently special attraction, has left in various pictures and sketches what may be called a veritable type of the figure of possession, "so faithful to nature that under all its aspects it remains true, so that now at the distance of more than two centuries, the undeniable signs of a nervous affection then entirely misunderstood, can be recognised." The most forcible of these works is the large painting at Vienna of "St. Ignatius Healing the Possessed," and in this the artist is undoubtedly open to the charge of marring artistic harmony by the naturalistic representation of morbid and repulsive phenomena. Both figures of the possessed show in the minutest details the characteristics of a violent attack of hysteria. The relieving feature in the violent convulsive attitudes assumed is the overwhelming reality of the figures, which lends their wild movements an air of irresistible necessity, and lessens the almost unavoidable theatrical element.

* "Les Démoniaques dans l'Art," page 25.

† "The Anatomy and Philosophy of Expression," by Sir Charles Bell, page 161.

